# Deciphering the Relationship Between Cycloheximides Structures and Their Different Biological Activities

**DOI:** 10.3389/fmicb.2021.644853

**Published:** 2021-04-07

**Authors:** Hang Thi Thu Nguyen, Jae Deok Kim, Vinit Raj, In Min Hwang, Nan Hee Yu, Ae Ran Park, Jung Seob Choi, Jintae Lee, Jin-Cheol Kim

**Affiliations:** ^1^Department of Agricultural Chemistry, College of Agriculture and Life Science, Institute of Environmentally Friendly Agriculture, Chonnam National University, Gwangju, South Korea; ^2^Eco-Friendly and New Materials Research Group, Korea Research Institute of Chemical Technology, Daejeon, South Korea; ^3^School of Chemical Engineering, Yeungnam University, Gyeongsan, South Korea; ^4^Hygienic Safety and Analysis Center, World Institute of Kimchi, Gwangju, South Korea

**Keywords:** antifungal activity, cycloheximides, phytotoxicity, molecular docking, streptomyces

## Abstract

*Streptomyces* species are the most important sources of antibacterial, antifungal, and phytotoxic metabolites. In this study, cycloheximide (CH) and acetoxycycloheximide (ACH) were isolated from the fermentation broth of *Streptomyces* sp. JCK-6092. The antifungal and phytotoxic activities of the two compounds (CH and ACH) and a cycloheximide derivative, hydroxycycloheximide (HCH), were compared. CH exhibited the strongest antagonistic activity against all the true fungi tested, followed by ACH and HCH. However, both CH and ACH displayed similar mycelial growth inhibitory activities against several phytopathogenic oomycetes, and both were more active than that of HCH. Disparate to antifungal ability, ACH showed the strongest phytotoxic activity against weeds and crops, followed by HCH and CH. ACH caused chlorophyll content loss, leaf electrolytic leakage, and lipid peroxidation in a dose-dependent manner. Its phytotoxicity was stronger than that of glufosinate-ammonium but weaker than that of paraquat in the *in vitro* experiments. CH and its derivatives are well-known protein synthesis inhibitors; however, the precise differences between their mechanism of action remain undiscovered. A computational study revealed effects of CHs on the protein synthesis of *Pythium ultimum* (oomycetes), *Magnaporthe oryzae* (true fungus), and *Capsicum annum* (plant) and deciphered the differences in their biological activities on different targets. The binding energies and conformation stabilities of each chemical molecule correlated with their biological activities. Thus, molecular docking study supported the experimental results. This is the first comparative study to suggest the ribosomal protein alteration mechanisms of CHs in plants and fungi and to thus show how the protein inhibitory activities of the different derivatives are altered using molecular docking. The correlation of structures features of CHs in respect to bond formation with desired protein was revealed by density functional theory. Overall collective results suggested that CHs can be used as lead molecules in the development of more potent fungicides and herbicides molecules.

## Introduction

Bioherbicides are weed control products derived from living organisms such as plants or microbes (McDade and Christians, 2000; Bailey et al., 2013; Bailey, 2014). Various herbicides are secondary metabolites produced by soil microbes. Cycloheximide (CH) was first isolated from *Streptomyces griseus* in 1949 and performed as an antifungal agent (Sisler and Siegel, 1967). The antifungal activity of various derivatives of CH was also reported. Two derivatives, acetoxycycloheximide (ACH) and streptovitacin-A [also named as hydroxycycloheximide (HCH)], showed less toxic to *Saccharomyces pastorianus* cells but higher toxic to cell-free protein-synthesizing system (Siegel et al., 1966). Furthermore, CH and HCH produced by *Streptomyces anulatus* also reported as herbicidal chemicals which displayed strong phytotoxicity against broadleaf weeds (Bo et al., 2019). HCH (a derivative of CH with a hydroxy group attached to C13 of cyclohexanone) which has previously been isolated from the culture broth of *S. griseus*, exhibited antifungal and antitumor activities (Kondo et al., 1990). Acetoxy cycloheximide (ACH; a derivative of CH with an acetoxy group attached to C13 of cyclohexanone) is a protein inhibitor and has also been shown to affect memory (Cohen and Barondes, 1968).

It was found that CH is a potent inhibitor of protein synthesis and acts by blocking translational elongation (Siegel and Sisler, 1963). A previous paper demonstrated that CH and lactimodomycin could block the translocation step in elongation through binding to the E-site of the large ribosomal subunit in eukaryotes. Both chemicals bind to the site between C3993 at the base of hairpin 88 of the 28S rRNA, between the 38th amino acid of L27a, and proline 54 of L36a in yeast, resulting in the blockage of eEF2-mediated tRNA translocation (Schneider-Poetsch et al., 2010). A previous study (Park et al., 2019) pointed out that an additional substituent at C13 of HCH enhanced translation inhibition compared to CH in human cells. Although a previous study (Schneider-Poetsch et al., 2010) postulated a mechanism of action of CH in human tumors, the precise reason why CH and its derivatives show different protein synthesis inhibition abilities in fungi and plants remains unknown.

Molecular docking is a structure-based drug tool that stimulates the molecular interaction and predicts the binding mode and affinity between ligands and receptor proteins (Meng et al., 2011). In recent years, this method has become the most common approach for discovery of new drugs or finding out the action mechanism of ligands. In our research, molecular docking was used to interpret the biological activities of CHs and lay the foundation for the development of new pesticides using CHs as lead molecule.

One *Streptomyces* sp. JCK-6092 isolated from soil was found to exhibit a strong *in vitro* antifungal activity after a screening of over 1,000 actinomycetes. During an *in vivo* experiment of dollar spot disease on creeping bentgrass caused by *Sclerotinia homoeocarpa*, the culture filtrate of the strain caused necrosis symptoms on creeping bentgrass leaves. Hence, in this study, we isolated and determined the chemical structures of the antifungal and phytotoxic metabolites from the fermentation broth of strain JCK-6092. CH and its derivative, ACH, were isolated from the culture broth of the actinomycete strain JCK-6092. Although CHs has been found since 1990s, their phytotoxic and antifungal activities have not previously been compared.

Therefore, this study aimed to (1) to isolate and identify the antifungal and phytotoxic metabolites from *Streptomyces* sp. JCK-6092, (2) to assess the phytotoxic and antifungal activities of the two compounds isolated from *Streptomyces* sp. JCK-6092, CH and ACH, as well as HCH, and (3) decipher the relationship between the chemical structures of the three CHs and the biological activities using molecular docking and density functional theory (DFT).

## Materials and Methods

### Strains and Cultural Conditions

To determine the antifungal activity of the three CHs, 18 true fungi and oomycetes were used; *Fusarium graminearum* (fusarium head blight on wheat), *F. oxysporum* f. sp. *cucumerinum* (fusarium wilt on cucumber), *F. o*. f. sp. *raphani* (fusarium wilt on radish), *Botrytis cinerea* (gray mold on tomato), *S. homoeocarpa* (dollar spot on creeping bentgrass), *Valsa ceratosperma* (valsa canker on apple), *Rhizoctonia solani* (brown patch on rice), *Curvularia lunata* (blight on zoysiagrass), *Endothia parasitica* (blight on chestnut), *Colletotrichum horii* (anthracnose on pepper), *Magnaporthe oryzae* (blast on rice), *Gaeumannomyces graminis* (take-all root rot on turf grass), *Magnaporthiopsis poae* (summer patch on Kentucky grass), *Botryosphaeria dothidea* (botryosphaeria canker on apple), *Phytophthora cactorum* (Phytophthora stem rot on apple), *Phytophthora cambivora* (Phytophthora collar rot on apple), *Phytophthora cinnamomi* (root rot on apple), and *Pythium ultimum* (damping off on cucumber). The growth of the plant pathogenic fungi was assessed using the method outlined in our previous report (Nguyen et al., 2020).

The actinomycete strain JCK-6092 was grown on Bennett’s agar and subsequently incubated in 5 mL of GSS broth containing soluble starch, 10 g L^–1^; glucose, 20 g L^–1^; soybean meal, 25 g L^–1^; yeast extract, 4 g L^–1^; beef extract, 1 g L^–1^; NaCl, 2 g L^–1^; K_2_HPO_4_, 0.25 g L^–1^; and CaCO_3_, 2 g L^–1^, pH 7.2. After incubation at 28°C for 3 days in an orbital incubator shaker at 180 rpm, this pre-culture was used to inoculate (1%) 200 mL of the same culture medium and cultivated for 7 days to isolate the antifungal and phytotoxic compounds.

### Identification of *Streptomyces* Strain

Identification of JCK-6092 was performed using 16S rRNA gene sequencing as conducted by Le et al. (2020), with slight modifications. Briefly, PCR amplification was performed using specific primer pair sets for the subsequent sequencing of 16S rRNA (Genotech Co., Daejeon, South Korea). PCR was performed under the following parameters: initial denaturation at 95°C for 5 min, 35 cycles of 95°C for 30 s, 58°C for 30 s, and 72°C for 50 s, followed by a final extension at 72°C for 5 min. The gene sequence was submitted to the Genbank database for Blast analysis. The phylogenetic tree was constructed using the neighbor-joining method in MEGA version 6.0 with 10,000 bootstrap replicates.

### Isolation and Identification of Active Metabolites

To isolate the active metabolites for subsequent analysis of phytotoxic and antifungal activities, the cell-free culture supernatant (1 L) of JCK-6092 was partitioned twice with EtOAc, and the organic solvent layer was then evaporated using a rotary vacuum evaporator (N-1110, EYELA Co., Tokyo, Japan). The resulting crude extract (0.49 g) was loaded onto a silica gel column (300 g, 70–230 mesh, 4 cm i.d. × 50 cm; Merck, Darmstadt, Germany) which was eluted with Chloroform/MeOH (96:4, v/v). The active fractions were combined and loaded onto the Sep-pak C18 Cartridge column (10 g, Vac 35 cc, Waters Corporation, MA, United States) and then eluted using a stepwise methanol gradient (0, 20, 40, 60, 80, and 100%, v/v), yielding compounds *1* (25 mg), and *2* (45 mg).

### Structural Confirmation of Isolated Active Metabolites

Liquid chromatography-electrospray ionization-mass spectrometry (LC-ESI-MS) analysis was performed with an LC-10AD pump (Shimadzu, Kyoto, Japan) with Sunfire C18 column (100 Å, 5 μm, 4.6 × 250, Waters Corporation) and API2000 mass spectrometer (AB SCIEX, Foster City, CA, United States). The purified compounds (1 mg) were separately dissolved in 200 μL MeOH to attain a final concentration of 5 mg mL^–1^. An aliquot (10 μL) of each solution was then injected into the instrument.

The ^1^H-NMR and ^13^C-NMR data of the purified compounds were recorded using Bruker Avance III HD 500 MHz instrument (Bruker Biospin GmbH, Rheinstetten, Germany) in chloroform-d (Cambridge Isotope Laboratories, Inc., MA, United States), and tetramethylsilane (TMS) was used as an internal standard in the NMR analysis (Nguyen et al., 2020).

### Assessment of *in vitro* Antifungal Activities of Cycloheximides

The antifungal activities of CHs and its derivatives ACH and HCH, which were isolated from the culture filtrate of *Streptomyces anulatus* strain-329 (Bo et al., 2019), were determined using the broth dilution method (Nguyen et al., 2020). The three chemicals were dissolved in acetone at a concentration of 10 mg mL^–1^ as a stock solution and were treated in a range of 0.012–100 μg mL^–1^. The final concentration of acetone was 1% v/v, and 1% acetone was used as an untreated control. All the plates were incubated for 5–7 days at 25°C. The minimum inhibitory concentration (MIC) values were measured as the lowest concentration at which the mycelial growth of test fungi was completely inhibited.

### Estimation of Cycloheximide Phytotoxicity

The phytotoxicity of three chemicals was evaluated by observation of the physiological symptoms of nine crops and 10 weeds, according to previous procedures (Bo et al., 2019). CH and its derivatives were diluted at 100 and 50 μg mL^–1^ containing 0.1% Tween 20 and sprayed on the leaves of 14-days-old weeds and plants. The phytotoxicity was recorded at 7 and 14 days after treatment (DAT), based on the physiological and morphological symptoms. Where 0 = no activity, 10–30 = slight activity, 40–60 = moderate activity, 70–90 = strong activity, and 100 = complete death.

### Chlorophyll Determination

Acetoxycycloheximide (ACH), which showed the strongest phytotoxicity in the whole-plant experiments, was tested for its effect on the chlorophyll content of cucumber plants compared with glufosinate and paraquat (Bo et al., 2019). In brief, leaf disks (1 g fresh weight) of cucumber plants were transferred to Falcon tubes containing 7 mL of distilled water. The water was removed after vacuum infiltration, and treatment solutions were added to the tubes. After incubation in the growth chamber for 36 h, the chlorophyll in each sample was extracted and total chlorophyll contents were calculated using the following formula:

$$\text{Chlorophyll}\,\left(\mathrm{mg}\,L^{-1}\right)=20.2^{*}\,A_{646\,\text{nm}}+8.02^{*}\,A_{663\,\text{nm}}$$

The experiment was repeated in triplicate.

### Cellular Leakage

The effect of ACH, glufosinate, and paraquat on cellular leakage was determined using leaf tissues of 7 days-old *Crocus sativus* seedlings (Bo et al., 2019). The leaf disks were placed in Petri dishes containing ACH, glufosinate, and paraquat at different concentrations and incubated in a growth chamber for 12 h. Electrolyte leakage was measured using an electronic conductivity meter (Denki Kagaku Co., Ltd., Mushashino, Japan). The experiment was performed in triplicate.

### Lipid Peroxidation

Lipid peroxidation was assessed by measurement of the amount of malondialdehyde (MDA), the most mutagenic product of lipid peroxidation (Bo et al., 2019). In brief, *C. sativus* leaf disks incubated with ACH, glufosinate, and paraquat solutions were homogenized in 0.5% thiobarbituric acid (TBA) and 20% trichloroacetic acid. The supernatants from homogenized solutions were then heated at 95°C for 25 min. The concentration of MDA in the harvested supernatants were measured using spectrophotometry. This experiment was conducted with three replicates.

### Homological Modeling of Three-Dimensional Protein Structures

Due to the different activities of three CHs to different targets, we investigated the binding interaction of CHs with three 60S ribosomal proteins of fungal *M. oryzae*, oomycete *P. ultimum*, and plant *Capsicum annuum*, respectively. Previous study reported that CHs bind with ribosomal protein, lead to inhibit translation elongation (Benkert et al., 2011). Even, 3D structures of these ribosomal proteins have not been yet reported in the protein data bank. Owing to the more than 71% similarities of the ribosomal proteins of *C. annuum*, *P. ultimum* and *M*. *oryzae* with 60 ribosomal proteins, in this study, these proteins were built using the homology modeling to investigate the good conformation stability of CHs with ribosomal proteins. *C. annuum* L44 sequence (PHT62729), *P. ultimum* L44 (UP000019132), and *M. oryzae* L44 (UP000009058) were retrieved from the National Center for Biotechnology Information (NCBI) and UniProt. These amino acid sequences were used for homological modeling using Schrodinger (Subhani et al., 2015) and the Swiss model to create the three-dimensional protein structure for estimation molecular docking interactions (Bienert et al., 2017), because three-dimensional structures of these proteins are not available in protein data bank format. In this study, homological modeling of the ribosomal protein of *C. annuum*, 60S ribosomal protein L44E templet (Gogala et al., 2014) was used, as the ribosomal protein of *C. annum* has 94.29% identity with the 60S ribosomal protein L44E templet. For the ribosomal protein of *P. ultimum*, templet 60S ribosomal protein L42-A (Ben-Shem et al., 2011) was selected because ribosomal amino acid residues od of *P. ultimum* has the 71.43% identity with templet 60S ribosomal protein L42-A. In addition, the ribosomal protein of *M*. *oryzae* was homologized, which has 81.13% identity with templet 60S ribosomal protein L42-A (Ben-Shem et al., 2011). These fungi and plant proteins were further analyzed using Ramchandran plots (Supplementary Figure 1).

### Three-Dimensional Structure Validation of Ribosomal Proteins

To evaluate the quality of the model, it is essential to know whether the built-up protein is a good quality model. The validity of the structures was therefore analyzed using Ramachandran plots (Hollingsworth and Karplus, 2010; Gore et al., 2017) to verify the most favored region (Supplementary Figure 1). As shown in Supplementary Figure 1A, it was observed that all built-up protein structures showed 93.2% of their residues in the more favorable region, 6% in additional allowed regions, and only 0.8% in disallowed regions. Meanwhile, as shown in Supplementary Figure 1B, 97% of residues are in a more favorable region and 2.5% in additional allowed regions, and 0.5% in the disallowed regions. Supplementary Figure 1C, showed 97.2% residues are in a more favorable region and 2.4% in additional allowed regions, and 0.4% in the disallowed regions. Consequently, this observation suggested that the homological models of these built proteins are good quality of three-dimensional protein structures. Also, quality of model was confirmed utilizing the local quality as well as a local quality score by the Swiss model (Benkert et al., 2011). Overall, all homology proteins showed the local quality score in the range of 0.8–1.0 (Supplementary Figure 1). And these built-up proteins are in a considerable range of local quality scores.

### Protein Structure Preparation and Ligand Collection

The ribosomal protein built structures were used for molecular docking studies. The active site was determined using the CASTp server^1^ and a predicted active pocket was assigned as a the grid to the virtual screening of molecules (Supplementary Figure 2; Tian et al., 2018). To refine the protein targets, protein preparation wizards’ approaches were used, using the Schrodinger suite. The appropriate numbers of hydrogen atoms were added, and protein was neutralized as per the Schrodinger standard procedure. Conversely, structures of CH, HCH, and ACH and two positive controls (lactimidomycin, and phyllanthoside; Park et al., 2019) were drawn by Chemdraw 12.0. For further optimization of the chemical structures of targeted molecules, the DFT method was adopted (El-Azhary and Suter, 1996). Later, the optimized molecules were used for docking with targeted proteins.

### Virtual Screening for Determining the Binding Energies Between the Ligands and Assigned Proteins

In the virtual screening, ligands were treated as rigid entities, while the receptor was treated as a flexible entity. To ensure results as valid, reliable, and reproducible, virtual screening studies were performed using AUTODOCK (Goodsell et al., 1996), and VINA (Seeliger and de Groot, 2010; Table 1). All molecules were analyzed through cluster analysis with eight runs. More stable conformations of docking complexes between ligands and proteins were selected for visualization of the interaction between amino acid residues of proteins and ligands by BIOVIA Discovery Studio Visualizer (Supplementary Figure 3). The total number of hydrogen and π–π interaction between the interacted amino acids with ligands was investigated (Supplementary Table 1).

**TABLE 1:** Molecular docking binding affinities of cycloheximide, hydroxycycloheximide, and acetoxycycloheximide, lactimidomycin, and phyllanthoside with the ribosomal proteins of *Magnaporthe oryzae, Pythium ultimum*, and *Capsicum annum* using AUTODOCK and VINA, respectively.

Compounds	*Magnaporthe oryzae* (kcal/mol)		*Pythium ultimum* (kcal/mol)		*Capsicum annum* (kcal/mol)	
	VINA	AUTODOCK	VINA	AUTODOCK	VINA	AUTODOCK
Cycloheximide	−5.26	−6.74	−5.54	−6.38	−5.03	−5.44
Hydroxycycloheximide	−5.04	−5.95	−5.04	−5.76	−5.13	−6.00
Acetoxycycloheximide	−5.17	−6.72	−5.10	−6.36	−5.13	−5.56
Lactimidomycin	−6.42	−8.54	−6.98	−6.34	−6.71	−6.43
Phyllanthoside	−6.96	−5.49	−7.82	−8.15	−5.73	−5.66

### Molecular Stability Study of Cycloheximides by Density Functional Theory

To evaluate the stability of molecules, the previously described density functional theory method was used (Ali et al., 2020). The frontier molecular orbitals were used to estimate the highest occupied molecular orbital (HOMO) and lowest unoccupied molecular orbital (LUMO) of CH, HCH, and ACH. The energy gap between HOMO and LUMO was calculated according to Koopmans theorem and Parr approximation (Parr et al., 1999) by:

(a) $$\Delta \,\text{E}={\text{E}}_{\text{LUMO}}-{\text{E}}_{\text{HOMO}}$$

By using HOMO and LUMO associated energies, chemical potential (μ) and chemical hardness (η) were calculated using the equations: (Ali et al., 2020);

(b) $$\mu =\frac{\text{ELUMO}+\text{EHOMO}}{2}$$

(c) $$\,\eta =\frac{\text{ELUMO}-\text{EHOMO}}{2}$$

Further, the electrophilicity (ω) and electronegativity (χ) were calculated from the ionization potential (*I*), which is mostly defined as the −E_HOMO,_ and the electron affinity (*A*) is equal to −E_LUMO._

(d) $$\chi =\frac{I+A}{2}$$

(e) $$\omega =\frac{\mu \,2}{2\,\eta }$$

All the molecules were optimized over minimum energy levels using the B3LYP level of theory with acetone as a solvent by Gaussian 09 program suite. The higher energy gap between HOMO and LUMO indicates the higher stability of the molecule, while the η value shows the reactivity order of the molecule, in which the molecules have the larger η value shows less reactivity nature of the molecule compared to a small η value (Parr et al., 1999; Ali et al., 2020).

## Results and Discussion

### Identification of JCK-6092

Comparison of the 16S rRNA gene of JCL-6092 (MW073398) with sequences in the GenBank database revealed that the strain was a *Streptomyces* sp. strain with 99% similarity to *S. noursei*, *S. albulus*, and *S. yunnanensis.* A phylogenetic tree of JCK-6092 was subsequently constructed through the neighbor-joining method (Supplementary Figure 4). Recently, taxonomy delineation of Streptomyces mostly depends on 16S rRNA. However, classification and identification of this genus is very complicated, which may lead to misidentification. Here, the JCK-6092 strain could not be identified to the species level due to the close relationship between the three *Streptomyces* strains mentioned above.

### Isolation and Identification of Antifungal and Phytotoxic Metabolites From *Streptomyces* sp. JCK-6092

Two compounds (*1* and *2*) were purified from the EtOAc layer of *Streptomyces* sp. JCK-6092. LC-ESI-MS of these chemicals showed molecular ion peaks at m/z 299.5 ([M+H]^+^) for compound *1* and *m/z 340.4* ([M+H]^+^) for compound *2* (Supplementary Figure 5). Based on these data, the molecular formulae of compound *1* and *2* were inferred to be C_15_H_23_NO_14_ and C_17_H_25_NO_6_, respectively. By comparison of the ^1^H and ^13^C NMR spectra data with those of previous reports, compounds *1* and *2* were identified as CH and ACH, respectively (Supplementary Figure 6 and Supplementary Table 2; Liang et al., 2016; Bo et al., 2019). CH was first isolated from the culture filtrate of *S. griseus* by Leach et al. (1947). This compound has also been found in the culture of *S. noursei*, *S. albulus*, *S. yunnanensis* (Rao, 1960; Dolezilová et al., 1965; Zhang et al., 2003; Liang et al., 2016). ACH and HCH were normally isolated together with CH (Liang et al., 2016; Bo et al., 2019). We thus showed that the strain JCK-6092 can synthesize both CH and ACH.

### Estimation of the *in vitro* Antifungal Activity of CH and Its Derivatives

The *in vitro* antifungal activity of the three CHs was evaluated against eighteen phytopathogenic fungi and oomycetes using the broth dilution method. As shown in Table 2, CH showed the strongest antifungal activity and inhibited the mycelial growth of all the true fungi tested, with a MIC values ranging from 0.78 to 50 μg mL^–1^. In the case of oomycetes such as *P. cactorum*, *P. cambivora*, *P. cinnamomi* and *P. ultimum*, both CH and ACH showed very strong and similar antifungal activity, with MIC value ranging from 0.097–0.39 to 0.0244–0.39 μg mL^–1^, respectively. HCH also showed strong or moderate antifungal activity against oomycetes with MIC range of 6.25–25 μg mL^–1^. The presence of piperidine-2,6-dione and cyclohexanone as the basic structure is necessary for the efficiency of these compounds as antifungal metabolites. HCH and ACH bearing one more group attached to the C13 of CH’s structure displayed weak fungicidal activity against the true fungi tested. In contrast, oomycetes were more sensitive to ACH (MIC values less than 0.78 μg mL^–1^) than true fungi. This indicated that the presence of one more acetoxy group at C13 reduced the binding affinity of ACH in true fungi, but not in oomycetes. This may be due to the different protein chains of the 60S ribosome of oomycetes with true fungi. Among three chemicals, HCH showed the weakest antifungal activity. This suggested that the presence of a hydroxy group at C13 dramatically reduced the binding affinity of HCH in true fungi and oomycetes. On the other hand, HCH inhibited protein synthesis much more efficiently than CH in a human cell line (Park et al., 2019), with 50% inhibition concentration (IC_50_) values of 0.97 μM for HCH and 2.64 μM for CH. These results suggested that the binding affinity of the three CHs to the target site may be different according to the target organism.

**TABLE 2:** *In vitro* antifungal activity of cycloheximides against the mycelial growth of several phytopathogenic fungi.

Phytopathogenic fungi	MIC value (μg mL^–1^)		
	CH	HCH	ACH
*Fusarium graminearum*	12.5	>100	100
*Fusarium oxysporum* f. sp. *cucumerinum*	50	>100	100
*Fusarium oxysporum* f. sp.*raphani*	25	>100	>100
*Botrytis cinerea*	6.25	>100	>100
*Sclerotinia homoeocarpa*	25	n.i.	100
*Valsa ceratosperma*	25	n.i.	100
*Rhizoctonia solani*	6.25	n.i.	100
*Curvularia lunata*	6.25	>100	50
*Endothia parasitica*	6.25	>100	50
*Colletotrichum horii*	6.25	100	12.5
*Magnaporthe oryzae*	1.56	>100	12.5
*Gaeumannomyces graminis*	0.78	>100	12.5
*Magnaporthiopsis poae*	1.56	50	12.5
*Botryosphaeria dothidea*	0.78	25	1.56
*Phytophthora cactorum*	0.39	25	0.39
*Phytophthora cambivora*	0.39	25	0.78
*Phytophthora cinnamomi*	0.097	6.25	0.0244
*Pythium ultimum*	0.195	12.5	0.195

*MIC, minimum inhibitory concentration; n.i., no inhibition.*

### Evaluation of the Phytotoxic Activities of ACH and Its Derivatives

**FIGURE 1:**
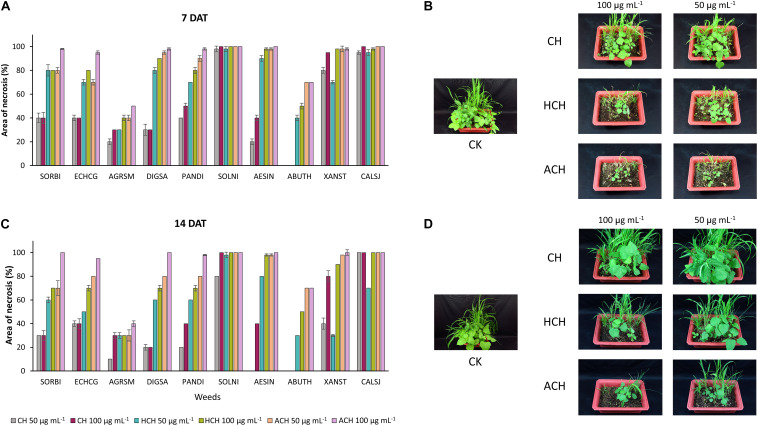
Phytotoxic effect of CH and its derivatives on weeds in post-emergence application at 7 in panel **(A,B)** and 14 days in panel **(C,D)** after treatment under greenhouse condition. Weeds were sprayed with CHs (50, or 100 μg mL^–1^). CH, cycloheximide; HCH, hydroxycycloheximide; ACH, acetoxycycloheximide; SORBI, *Sorghum bicolor*_;_ ECHCG, *Echinochloa crus-galli*; ARGSM, *Agropyron smithii*; DIGSA, *Digitaria sanguinalis*; PANDI, *Panicum dichotomiflorum*; SOLNI, *Solanum nigrum*; AESIN, *Aeschynomene indicia*; ABUTH, *Abutilon theophrasti*; XANST, *Xanthium strumarium*; CALSJ, *Calystegia japonica*. Each value represents the mean ± standard deviation of two runs, with three replicates per run.

The post-emergence phytotoxicity of CH and its derivatives against monocotyledon weeds [*Sorghum bicolor* (SORBI), *Echinochloa crus-galli* (ECHCG), *Agropyron smithii* (ARGSM), *Digitaria sanguinalis* (DIGSA), *Panicum dichotomiflorum* (PANDI)], and dicotyledon weeds [*Solanum nigrum* (SOLNI), *Aeschynomene indica* (AESIN), *Abutilon theophrasti* (ABUTH), *Xanthium strumarium* (XANST), *Calystegia japonica* (CALSJ)] is illustrated in Figure 1. All of the tested chemicals exhibited phytotoxicity at both 50 and 100 μg mL^–1^ against 10 kinds of weeds, 7 and 14 days after treatment (DAT) except CH that did not have any phytotoxicity against ABUTH. ACH displayed the strongest phytotoxicity with the phytotoxicity over 95% at 100 μg mL^–1^ at 14 DAT against the test weeds except for ARGSM (40%) and ABUTH (70%). HCH exhibited higher phytotoxicity than CH with the control values over 70% at 100 μg mL^–1^ at 14 days after treatment, except ARGSM and ABUTH, while the control value of CH at 100 μg mL^–1^ is over 20% (Figures 1C,D). Furthermore, CH at 50 μg mL^–1^ inhibited the growth of AESIN at 7 DAT (Figures 1A,B), but could not remain the effective activity at 14 DAT (Figures 1C,D).

**FIGURE 2:**
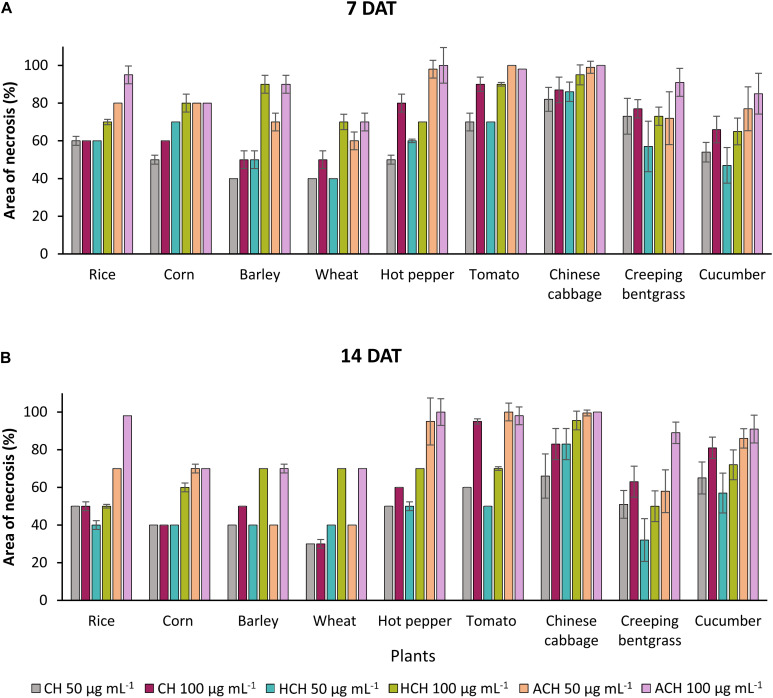
Phytotoxic effects of CHs and its derivatives on crops in the post-emergence application at 7 in panel **(A)** and 14 days in panel **(B)** after treatment under greenhouse condition. Plants were sprayed with CHs (50 or 100 μg mL^–1^). CH, cycloheximide; HCH, hydroxycycloheximide; ACH, acetoxycycloheximide. Each value represents the mean ± standard deviation of two runs with three replicates per run.

The three CHs also exhibited notable phytotoxicity in crops and turfgrass (Figure 2). As for the weeds, ACH presented the strongest phytotoxicity to most of the plants tested, followed by HCH and CH. However, CH exhibited stronger phytotoxicity than HCH in treatment with tomato, creeping bentgrass, and cucumber. Thus, the herbicidal activity of the three CHs could be summarized as follows: ACH > HCH > CH. A previous study also demonstrated that HCH was a more potent herbicide than CH (Bo et al., 2019). However, the phytotoxicity of ACH has not been reported in detail. The substituted position of hydroxy and acetoxy group on cyclohexanone may enhance the phytotoxicity.

### Effect of Acetoxycycloheximide on the Chlorophyll Content, Cellular Leakage, and Malondialdehyde Production of Cucumber Leaf

**FIGURE 3:**
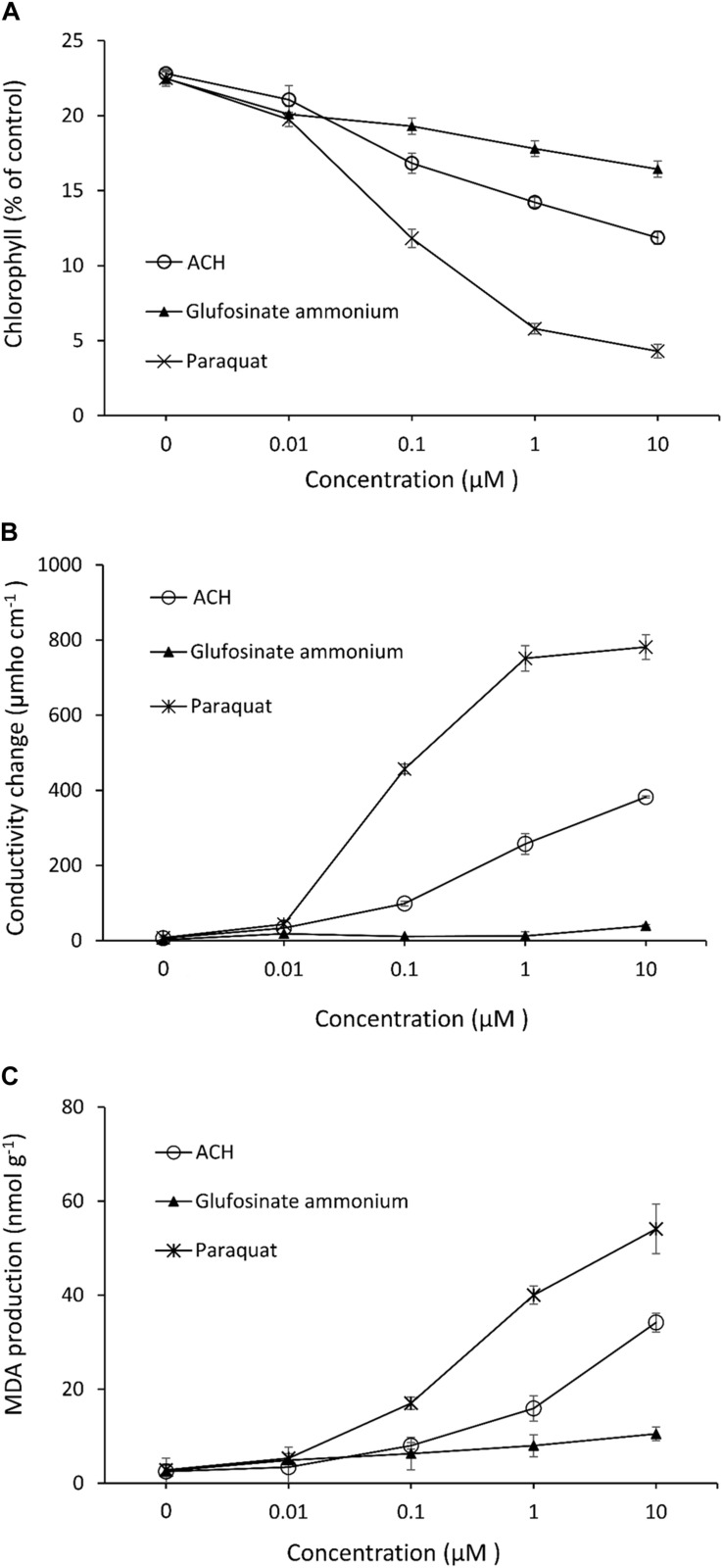
Effect of acetoxy-cycloheximide and paraquat, glufosinate-ammonium on **(A)** chlorophyll contents **(B)** cellular leakage and, **(C)** MDA production of cucumber cotyledon leaf squares. The tissues were exposed to continuous light at 120 mmol/m^2^/s PAR at 25°C for 24 h, following 12 h of dark incubation. Error bars are ± 1 SE of the means.

Acetoxycycloheximide, which showed the strongest phytotoxicity among the three CHs, was used to evaluate herbicide parameters with the two commercial products glufosinate-ammonium and paraquat. The chlorophyll loss, electrolyte leakage, and MDA production in the cucumber leaves were measured after treatment with different concentrations of each chemical. The chlorophyll contents decreased as the concentration of each treatment increased. Paraquat triggered the strongest loss of chlorophyll, reaching up to 80.93% inhibition at a concentration of 10 μM, while ACH and glufosinate-ammonium moderately reduced the chlorophyll content to 47.98, and 26.86%, respectively (Figure 3A). The effect of electrolytic leakage caused by ACH, paraquat, and glufosinate-ammonium in cucumber was determined by measuring the conductivity of the treatment solution. The results showed a significantly different degree of conductivity change between ACH, paraquat, and glusinate-ammonium. There was a vast leakage of electrolytes in the presence of paraquat (10 μM) of 781.3 μmho cm^–1^ while glucosinate-ammonium slightly changed the conductivity to 39.3 μmho cm^–1^ at 10 μM. AHC moderately affected the electrolyte leakage to 382.0 μmho cm^–1^ at a concentration of 10 μM (Figure 3B).

The production of MDA, which causes membrane lipid peroxidation of cucumber leaves treated with ACH, and the two control agents are shown in Figure 3C. The level of MDA treated with glufosinate (10 μM) was slightly increased to 10.5 nmol g^–1^ while the production level of MDA was 54.1, and 34.2 nmol g^–1^ in paraquat and ACH, respectively, both at a concentration of 10 μM.

Chlorophyll degradation or loss in leaves is usually used as an indicator of senescence or cell death in plants due to its visibility. Cellular leakage, triggering a loss of membrane integrity and MDA accumulation, and lipid peroxidation of the membrane are two common indicators that determine phytotoxicity levels. Based on these indices, paraquat is the most powerful herbicide, followed by ACH and glufosinate-ammonium. While paraquat acts as a photosynthesis inhibitor and glufosinate inhibits glutamate, ACH, together with other CHs, inhibits protein synthesis. Photosystem I (PSI) is disturbed by paraquat, leading to the more rapid destruction of cell membranes. Moreover, paraquat toxicity in the cell triggers lipid peroxidation (Chun et al., 2002), which explains why paraquat has such a strong effect on all indices. Glucosifate inhibits the enzyme glutamine synthetase from anabolizing glutamate and ammonia, thus leading to ammonia poisoning in the plant cells. Therefore, glufosinate caused no electrolyte leakage, which has also been noted in previous papers (Bo et al., 2019). ACH, a protein synthesis inhibitor, showed moderate activity compared to two the commercial herbicides. Protein is necessary for plant survival, growth and development, hence, protein synthesis inhibition affects the entire plant at the cellular level.

### Enzyme Inhibitory Activity of Substituted CHs Against Ribosomal Proteins Using Virtual Screening

To identify the binding interactions between the substituted CHs and the ribosomal proteins of *M. oryzae, P. ultimum*, and *C. annum*, two parallel virtual screening was performed using AUTODOCK and VINA approaches. For each ligand, a minimum eight-run clustered analysis was employed to determine the minimum binding conformation between the ligands and proteins. As a result, all molecules exhibited the binding energies with the targeted proteins above the −5.0 kcal/mol, which was comparable to the two positive controls (lactimidomycin, and phyllanthoside; Table 1). The random 24 runs showed a more stable binding confirmation for the substituted CHs (Supplementary Figures 3, 7).

**FIGURE 4:**
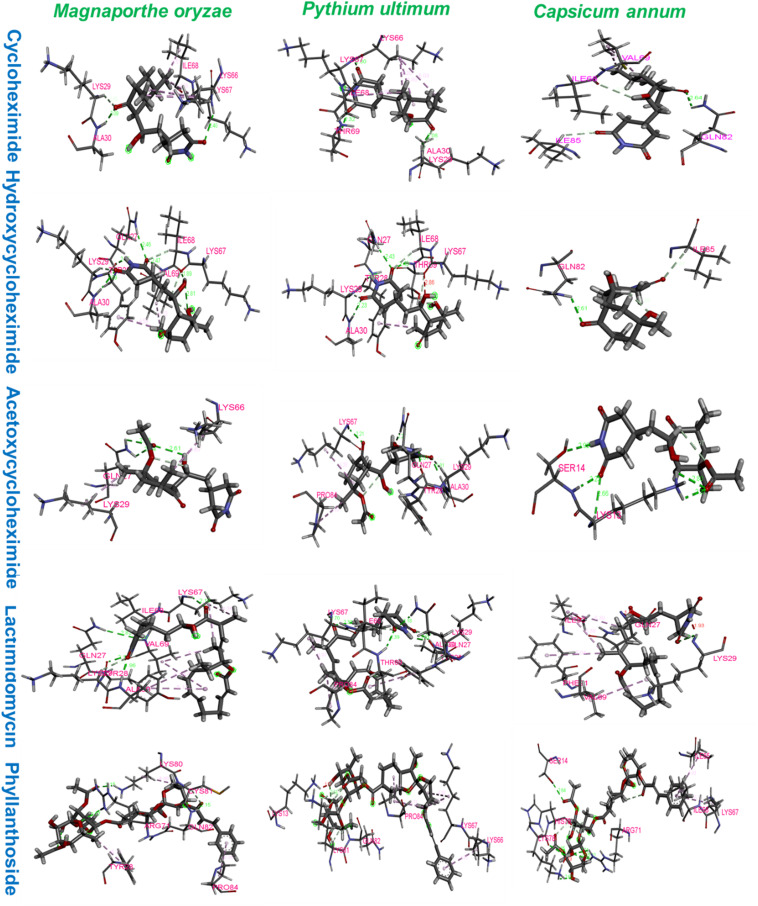
Amino acid residues interactions of ribosomal proteins of Magnaporthe oryzae, Pythium ultimum, and Capsicum annum with cycloheximide, hydroxycycloheximide, acetoxycycloheximide, lactimidomycin, and phyllanthoside, respectively, where lactimidomycin and phyllanthoside used as the positive control.

The ribosomal protein of *M. oryzae* showed better binding with CH compared to HCH and ACH. All of these compounds bound with the conserved amino acid residues Gln27, Lys29, and Lys66, which is similar to the binding mechanism of positive controls, which interacted with the Gln27 and Lys29 residues of the *M. oryzae* ribosomal protein. Therefore, it can be suggested that these amino acid residues may be essential for drug binding in the active pocket of the ribosomal protein of *M. oryzae* to produce the inhibitory effects against the ribosomal protein synthesis. Also, the binding affinities with the *M. oryzae* protein were slightly similar to that of *P. ultimum* during molecular docking, and the binding energies of CH were slightly higher than ACH and HCH. These molecules bind with the conserved amino acid residues Lys29, Ala30, and Lys67 of ribosomal protein of *P. ultimum*, in a similar binding interaction as seen with positive controls (amino acid residues Lys29, Ala30, and Lys67; Figure 4). However, the binding affinities in the molecular docking of ACH with *C. annum* were found to be higher than that of CH and more similar to HCH (Table 1 and Supplementary Table 1). The trend in binding energies with the ribosomal protein of *C. annum* was thus identified as ACH ≥ HCH > CH. The binding of these molecules and the positive controls show similar patterns with common amino residues (Ser14 and Ile85). It is well reported that the interaction between drugs and proteins is more important than the interacting forces with the amino acid residues of the protein (Xiao and Cushman, 2005). Therefore, we can conclude that CH, ACH, and HCH occupy the binding site of the ribosomal proteins of *M. oryzae, P. ultimum*, and *C. annum* and may thus suppress protein synthesis, resulting to inhibition of ribosomal protein synthesis. Thus, molecular modeling supported to the inhibition of the ribosomal protein synthesis mechanism. The number of hydrogen and π–π bonds between the ligands and proteins revealed that CHs bind strongly with ribosomal proteins, which help to inhibit the protein synthesis prolong. Interestingly, experimental biological activities and molecular docking patterns are similar, which supports the aim of the research to inhibit the ribosomal protein synthesis (Figures 1, 2 and Tables 1, 2).

### Structural Stabilities of CH, HCH, and ACH Determined by Density Functional Theory (DFT) Approaches

**FIGURE 5:**
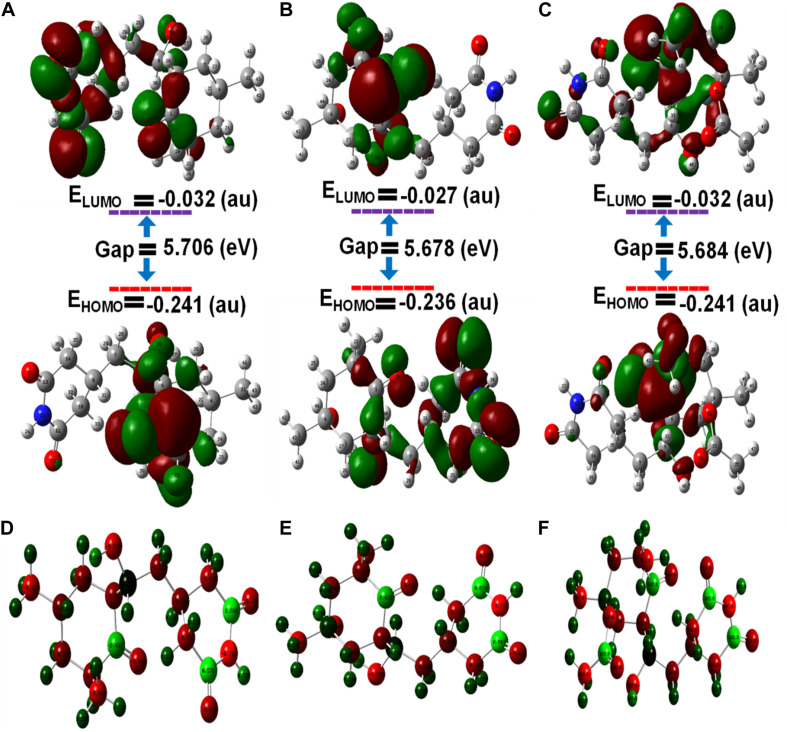
Optimized molecular geometries with **(A)** cycloheximide, **(B)** hydroxycycloheximide, and **(C)** acetoxycycloheximide were calculated using the DFT approach and structures were optimized with the charges and atomic numbers **(D–F)** of cycloheximide, hydroxycycloheximide, and acetoxycycloheximide. The optimized structures are labeled with an atom numbering.

The frontier molecular orbital’s (FMOs), namely the highest occupied molecular orbital (HOMOs) and lowest unoccupied molecular orbital (LUMOs), of ligands were estimated by density functional theory (DFT). The HOMO acts as an electron donator, while the LUMO acts as an electron acceptor (Teotia et al., 2019). Frontier molecular theory suggests that HOMO and LUMO are important factors affecting the biological properties, ionization, molecular reactivity, and electron affinity of molecules. Therefore, FMO studies can reveal significant insight into the biological mechanisms of active compounds. As shown, in Figure 5, CH, HCH, and ACH displayed the energy gaps of 5.70, 5.67, and 5.68 eV between HOMO and LUMO. Among them, CH had a slightly higher energy gap, and consequently shows higher stability. It is well reported that a higher energy gap is indicative of higher stability of the molecule, because higher energies are required to shift the electron from HOMO to LUMO (Balachandar et al., 2017; Joubert, 2019). Therefore, the molecules could be ranked in order of stability as CH > ACH > HCH.

As displayed in Supplementary Table 3 and Figure 5, in CH, the HOMO is located at the cyclohexanone motif and LUMO is mainly localized at cyclohexanone and piperidine-2,6-dione rings. In the HCH, the cyclohexanone and piperidine-2,6-dione rings displayed the HOMO, while the cyclohexanone ring contained the LUMO. In ACH, both HOMO and LUMO were displayed at cyclohexanone and piperidine-2,6-dione, excluding the dimethyl group. These observations indicate that the activity accompanying these molecules could usually be attributed to the cyclohexanone and piperidine-2,6-dione motifs, while the dimethyl group provides structural bulkiness or hydrophobic function. Thus, the cyclohexanone and piperidine-2,6-dione motifs are the main functional groups to interact with the amino acid residues of the active pocket of ribosomal protein of *M. oryzae, P. ultimum*, and *C. annum*, thus giving rise to biological activities. The nitrogen atoms of the optimized structures of CH, HCH, and ACH exhibited higher electronegative charges and the lowest atomic charge value (*N* = −0.773), while the oxygen of the cyclohexanone ring had a lower atomic charge compared to the oxygen atoms of the piperidine structure. Therefore, the oxygen of the cyclohexanone ring would favorably interact with the positively charged amino acid residues inside the ribosomal protein. These structural features of CHs molecules and a similar binding and activity pattern of computational and *in vitro* studies suggested that CHs are promising molecules for phytotoxic and antifungal activities.

## Conclusion

Cycloheximide and ACH were isolated from the culture broth of *Streptomyces* sp. JCK-6092. The phytotoxic and antifungal activities of CH and its derivatives ACH and HCH were studied in various weeds, crops, and phytopathogenic fungi. The three compounds showed different biological activities according to the target organisms tested, which included plants, true fungi, and oomycetes. The CHs showed promising binding affinity toward the E-site of the 60S ribosomes in molecular docking and the stability of CH and ACH in the DFT study helps to reveal the structure-activity correlation of molecules and also helps to understand the molecular mechanistic of the binding with ribosomal protein to inhibit the protein synthesis. Thus, the collective correlation between the molecular docking study and biological activities revealed that CH had the strongest antifungal activity against true fungi, whereas ACH exhibited the most potent phytotoxicity and similar antifungal activity to CH against oomycetes. This is thus the first comparative study, to our knowledge, to investigate the mechanisms of action of CHs against plants, fungi, and oomycetes and to use molecular docking to predict how the different derivatives may alter ribosomal protein synthesis by acting as antagonists. Therefore, these results may helpful in the development of more potent fungicides and herbicides.

## Data Availability Statement

The datasets presented in this study can be found in online repositories. The names of the repository/repositories and accession number(s) can be found in the article/Supplementary Material.

## Author Contributions

J-CK, JC, and JL designed this study. HN and NY isolated and identified strain JCK-6092. HN, IH, and JK isolated and identified CHs. HN, AP, and NY were performed *in vitro* antifungal bioassays. JK and JC performed *in vivo* and *in vitro* herbicidal experiments. HN and JK analyzed the antifungal and herbicidal data. VR and JL performed the molecular docking. HN, JL, and J-CK wrote and revised the manuscript. All authors read and approved the submitted version.

## Conflict of Interest

The authors declare that the research was conducted in the absence of any commercial or financial relationships that could be construed as a potential conflict of interest.

## Footnotes

1 http://sts.bioe.uic.edu/
