# Supplementary material for: Deciphering the Relationship Between Cycloheximides Structures and Their Different Biological Activities
Source: Front Microbiol. 2021 Apr 7;12:644853. doi: 10.3389/fmicb.2021.644853 (PMC8058199; doi:10.3389/fmicb.2021.644853)
Supplement: Supplementary file 1 [file Data_Sheet_1.docx]

Supplementary Material

# Supplementary Figures and Tables

## Supplementary Figures


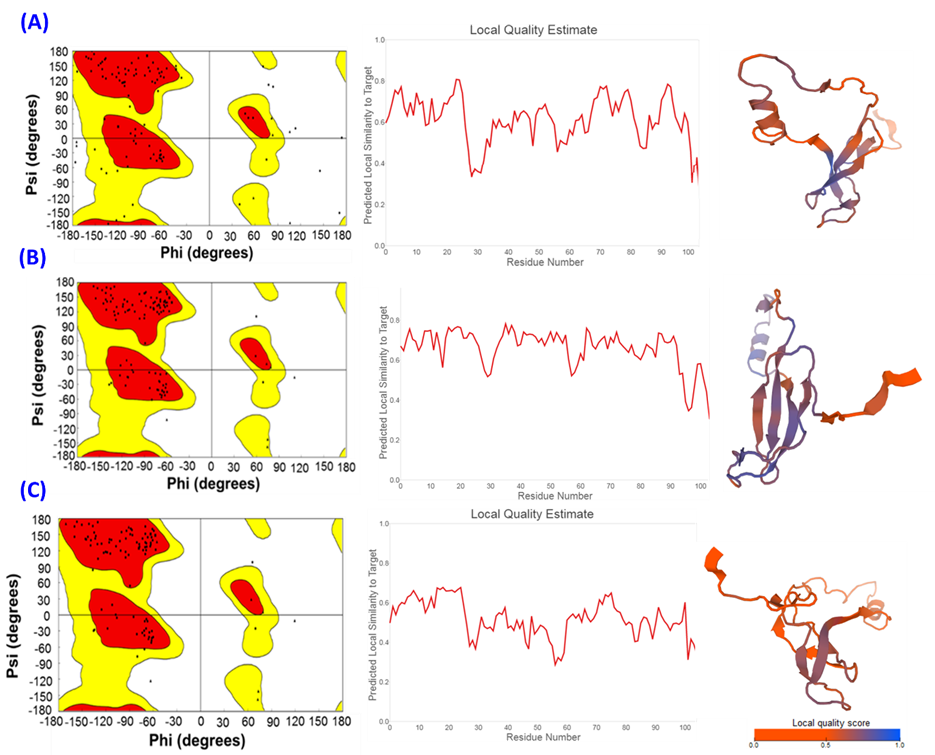


**Supplementary Figure S1.** Validation of ribosomal proteins of *Magnaporthe oryzae* (A)*, Pythium ultimum* (B), and *Capsicum annuum* (C) using Ramachandran plot, and qualitative model energy analysis (QMEAN) to determine the local quality score as shown by red to blue scale. Local quality estimation of these ribosomal proteins was evaluated between the predicted local similarity to target and residue number.


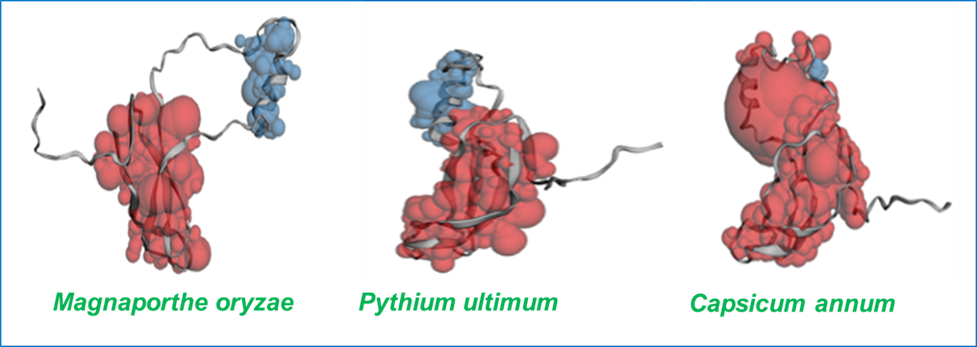
**Supplementary Figure 2.** Predicted binding active pockets, where red color and blue color show the active binding pockets ribosomal proteins of *Magnaporthe oryzae, Pythium ultimum*, and *Capsicum annuum*.


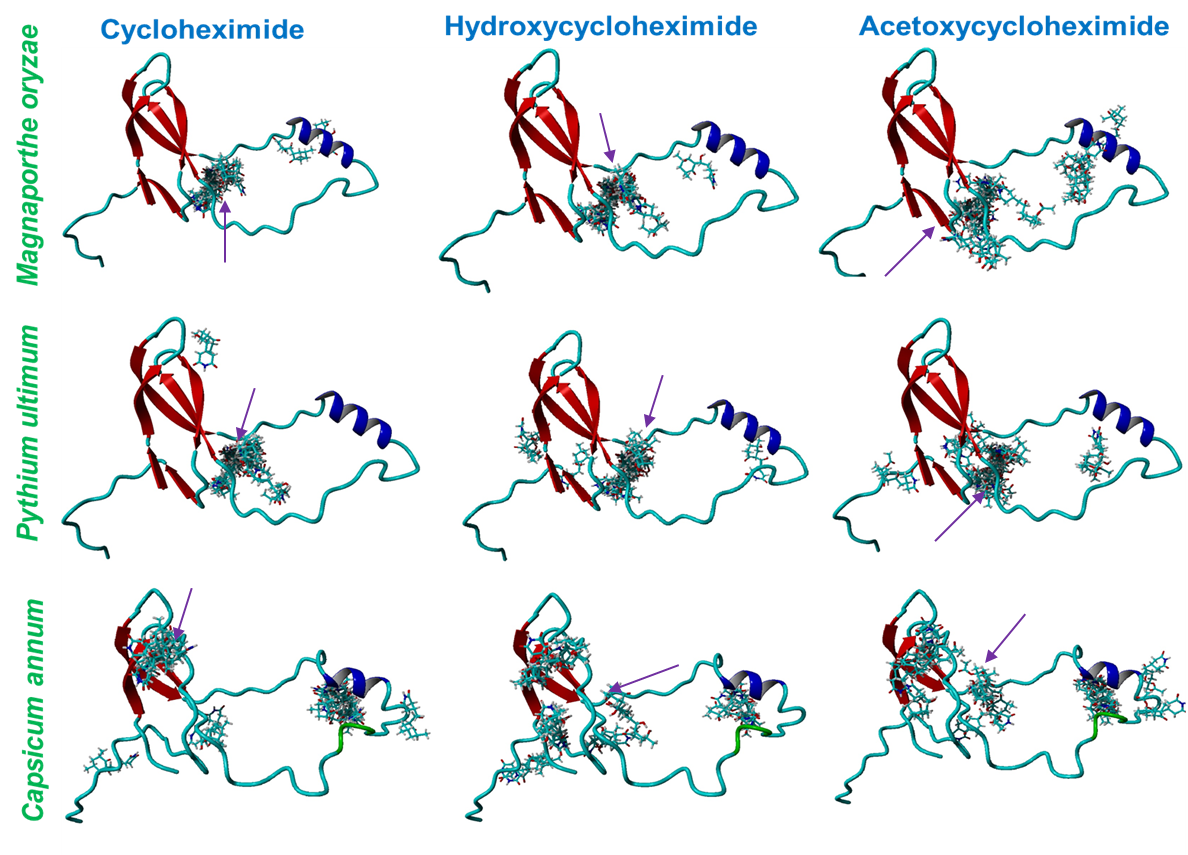


**Supplementary Figure S3.** Molecular binding conformational (25 runs of each molecule with assigned proteins) of cycloheximide, hydroxycycloheximide, and acetoxycycloheximide with entire ribosomal proteins of *Magnaporthe oryzae, Pythium ultimum,* and *Capsicum annum* using AUTODOCK, respectively are used to predict the best binding pocket with better stable conformation (marked by arrow).


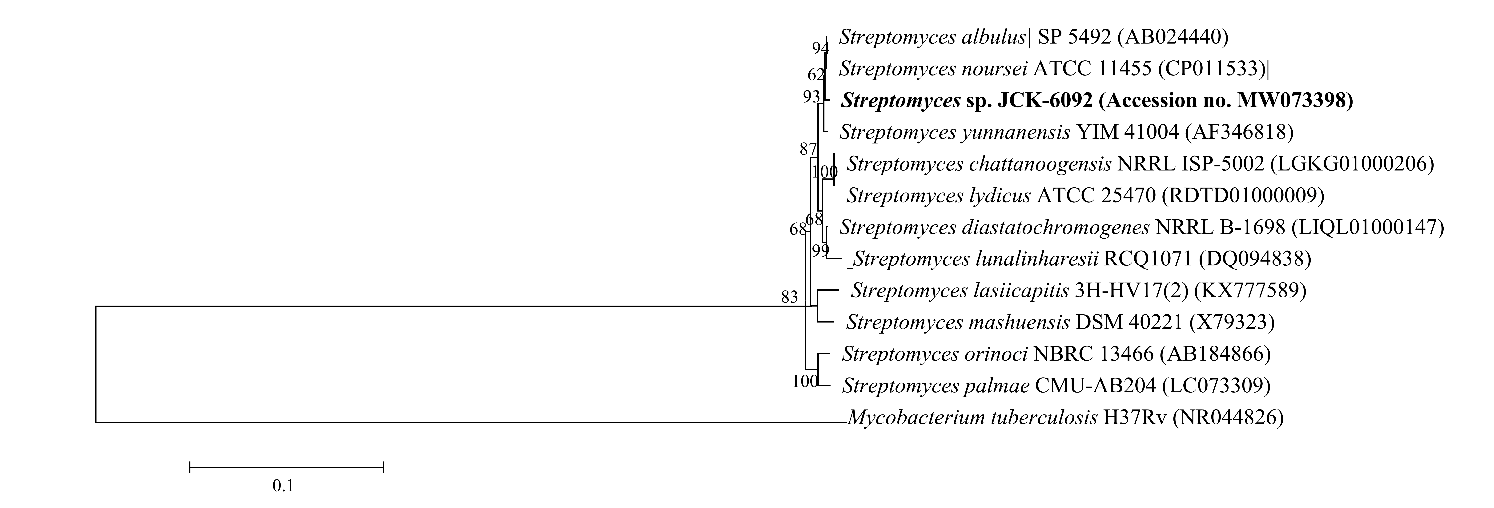


**Supplementary Figure S4.** Neighbour-joining tree based on the 16S rRNA gene sequence of JCK-6092. The evolutionary history was inferred using the Neighbor-joining method. The bootstrap consensus tree was inferred from 10,000.


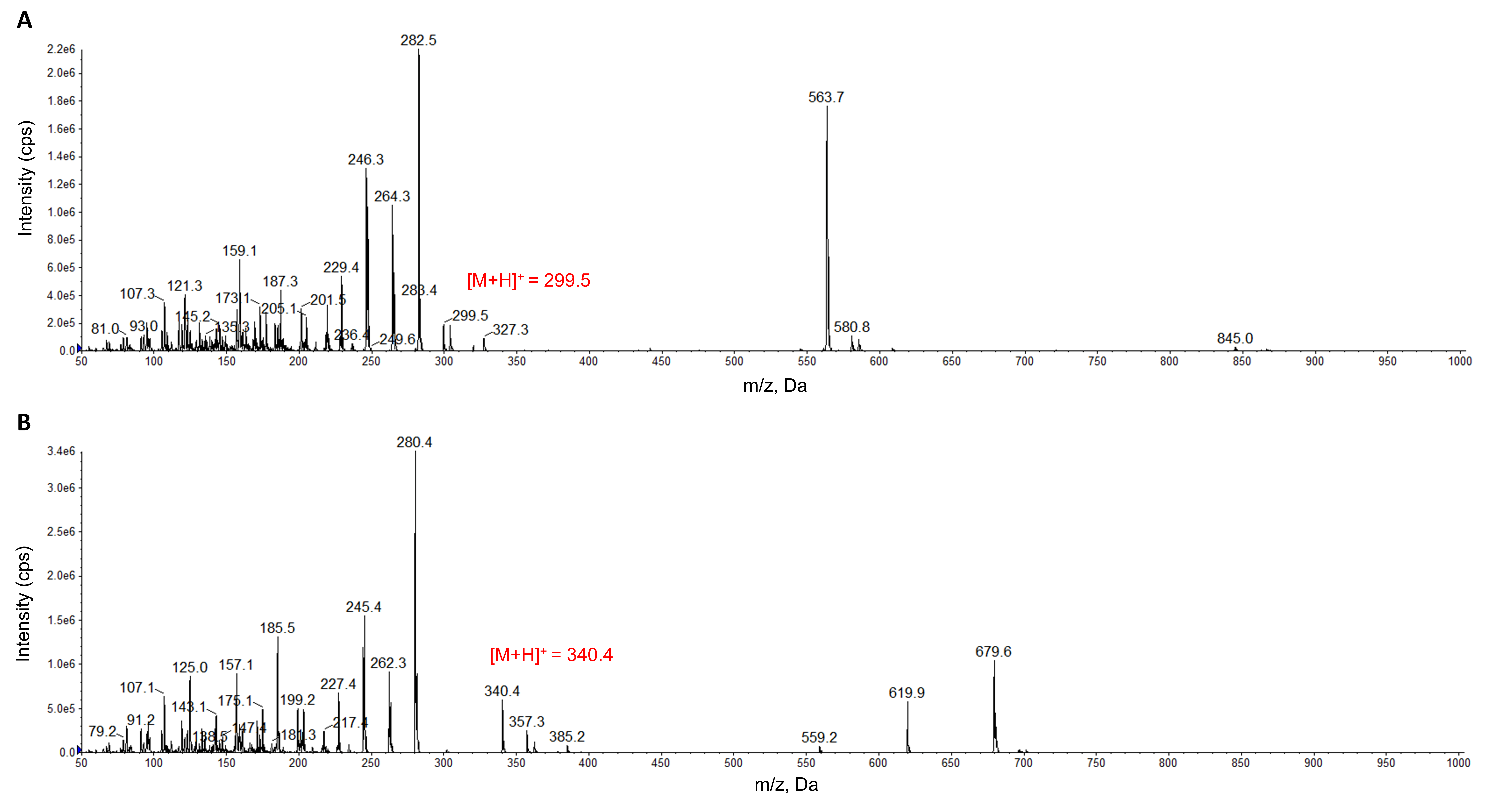


**Supplementary Figure S5.** LC-ESI(+)-MS/MS spectra of (**A**) compound **1** (cycloheximide) and (**B**) **2** (acetoxycycloheximide)


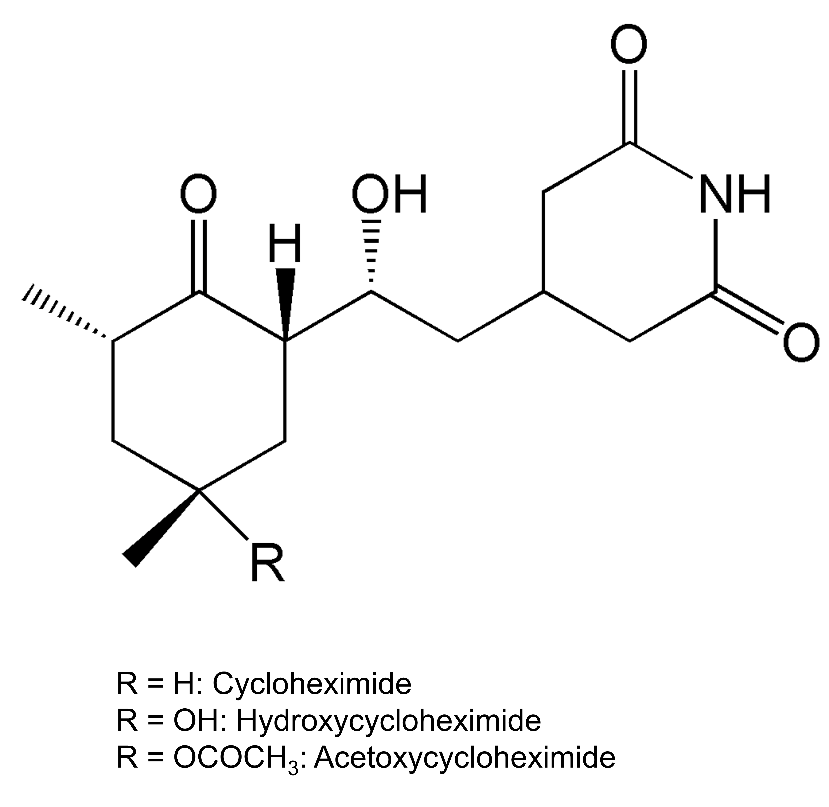


**Supplementary Figure S6.** Chemical structures of cycloheximide and its derivatives


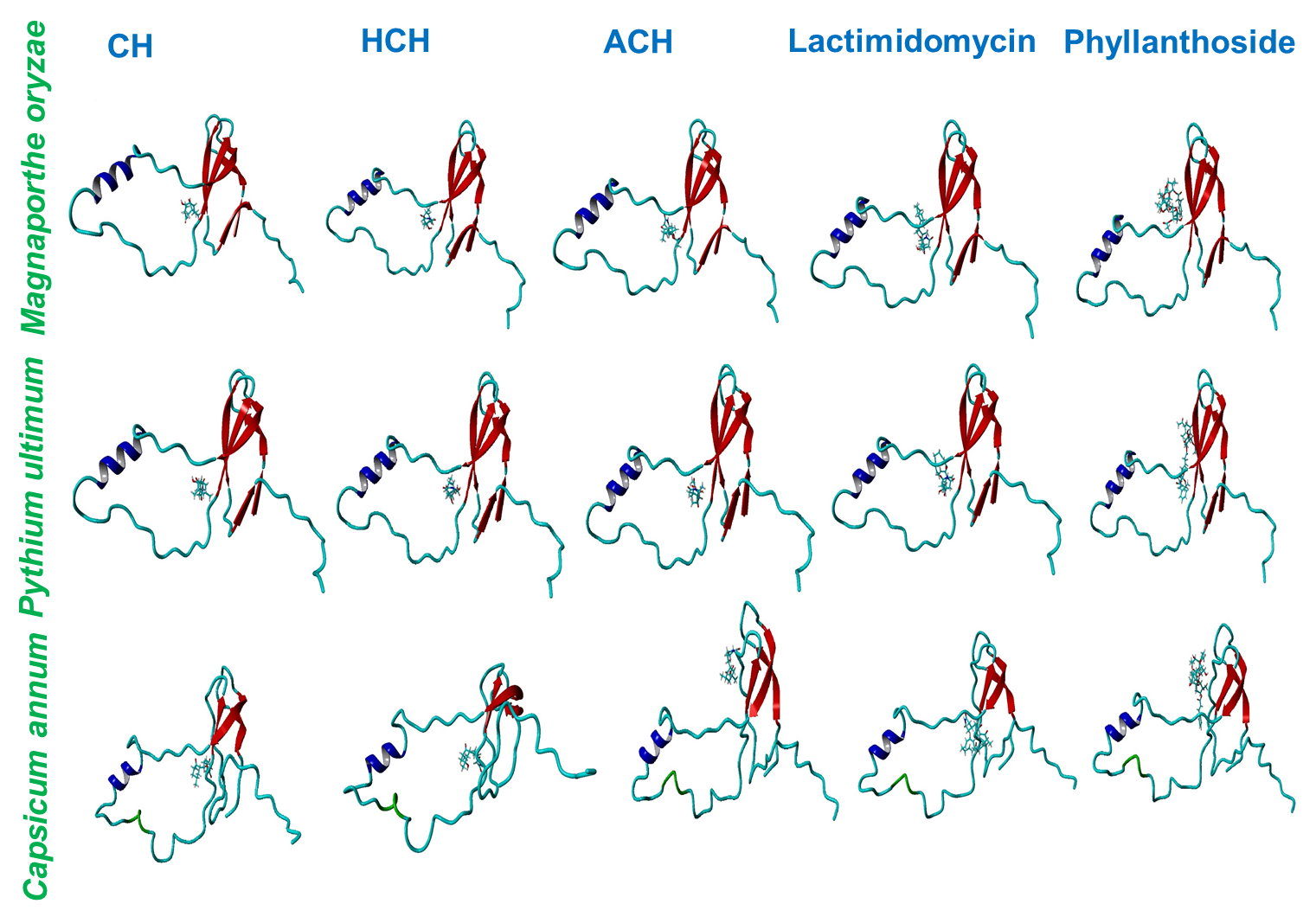


**Supplementary Figure S7**. Overall conformation interaction of cycloheximide, hydroxycycloheximide, and acetoxycycloheximide, lactimidomycin, and phyllanthoside with *Magnaporthe oryzae, Pythium ultimum*and, and *Capsicum annum*, respectively.

**Supplementary Table**

**Supplementary Table 1** Amino acids interaction and number of hydrogen and π-π between cycloheximide, hydroxycycloheximide, acetoxycycloheximide, lactimidomycin, phyllanthoside with the ribosomal proteins of *Magnaporthe oryzae, Pythium ultimum,* and *Capsicum annum*, respectively.

| **Compounds** | ***Magnaporthe oryzae*** | | ***Pythium ultimum*** | | ***Capsicum annum*** | |
| --- | --- | --- | --- | --- | --- | --- |
|  | **Bonds** | **Amino acids**  **interaction** | **Bonds** | **Amino acids**  **interaction** | **Bonds** | **Amino acids**  **interaction** |
| CH | 2H,5π | Lys29, Ala30, Lys66, Lys67, Ile68 | 3H,4π | Lys29, Ala30, Lys66, Lys67, Ile68, Thr69 | 1H,4π | Gln82, Ile68, Val69, Ile85 |
| HCH | 6H,5π | Gln27, Tyr28, Lys29, Ala30, Lys67, Ile68, Val69 | 3H,4π | Gln27, Tyr28, Lys29, Ala30, Lys67, Ile68, Thr69 | 1H,2π | Glyn83, Ile85 |
| ACH | 3H,3π | Gln27, Lys29, Lys66 | 4H,3π | Gln27, Tyr28, Lys29, Ala30, Lys67, Pro84 | 5H,1π | Lys13, Ser14 |
| Lactimidomycin | 4H,7π | Gln27, Lyr28, Lys29, Ala30, Lys67, Ile68, Val69 | 5H,3π | Gln27, Tyr28, Lys29, Ala30, Lys67, Ile68, Thr69, Pro84 | 1H,4π | Gln27, Lys29, Ile63, Val69, Phe91 |
| Phyllanthoside | 4H,4π | Tyr28, Arg71, Lys80, Cys81, Gln82, Pro84 | 2H,8π | Lys13, Lys66, Lys67, Lys81, Gln82 | 5H,6π | Ser14, Lys67, Ile68, Arg71, Lys78, His79, Ile85 |

**Supplementary Table 2 NMR spectroscopy data of cycloheximides and acetoxy cycloheximides**

|  | **Cycloheximide** | | **Acetoxycycloheximide** | |
| --- | --- | --- | --- | --- |
| Position | ^1^H^a^ | ^13^C^a^ | ^1^H^a^ | ^13^C^a^ |
| 2 |  | 172.17 |  | 172.15 |
| 3 | Ha: 2.31 (d, 1H), Hb: 2.77 (d, 1H) | 38.50 | Ha: 2.79 (d, 1H), Hb: 2.30 (d,1H) | 38.46 |
| 4 | 2.45 (m, 1H) | 27.54 | 2.45 (m, 1H) | 27.64 |
| 5 | Ha: 2.78 (d, 1H), Hb: 2.29 (d, 1H) | 37.18 | Ha: 2.76 (d, 1H), Hb: 2.30 (d, 1H) | 37.21 |
| 6 |  | 171.97 |  | 171.96 |
| 7 | Ha: 1.19 (m, 1H), Hb: 1.65 (m, 1H) | 37.81 | Ha: 2.11 (t, 1H), Hb: 2.49 (m, 1H) | 36.43 |
| 8 | 4.23 (tt, 1H) | 66.54 | 4.25 (m, 1H) | 66.54 |
| 9 | 2.51 (m, 1H) | 50.08 | 2.34 (dd, 1H) | 50.77 |
| 10 |  | 216.58 |  | 213.56 |
| 11 | 2.65 (m, 1H) | 40.54 | 2.55 (m, 1H) | 40.54 |
| 12 | Ha: 1.60 (dd, 1H), Hb: 1.90 (dd, 1H) | 42.57 | Ha: 1.19 (m, 1H), Hb: 1.61 (m, 1H) | 37.77 |
| 13 | 2.23 (m, 1H) | 26.70 |  | 79.95 |
| 14 | Ha: 1.81 (m, 1H), Hb: 1.92 (m, 1H) | 32.97 | Ha: 2.53 (d, 1H), 1.75 (d, 1H) | 45.39 |
| 15 | 1.26 (d, 3H) | 18.36 | 1.05 (d, 3H) | 14.03 |
| 16 | 1.01 (d, 3H) | 14.17 | 1.83 (s, 3H) | 22.29 |
| 17 |  |  |  | 170.30 |
| 18 |  |  | 2.00 (s, 3H) | 22.37 |
| NH | 7.95 (s) |  | 8.06 (s) |  |
| OH |  |  |  |  |

^a^ 125 MHz

^b^ 500 MHz

## Supplementary Table 3. DFT calculation results of cycloheximide, hydroxycycloheximide, and acetoxycycloheximide for quantum chemical parameters

| **Quantum chemical parameters (eV unit)** | **Cycloheximide** | **Hydroxycycloheximide** | **Acetoxycycloheximide** |
| --- | --- | --- | --- |
| HOMO | -6.57 | -6.42 | -6.56 |
| LUMO | -0.87 | -0.74 | -0.88 |
| Energy gap (ΔE) | 5.70 | 5.67 | 5.68 |
| Chemical potential (µ) | -3.72 | -3.58 | -3.72 |
| Electron affinity (A) | 0.87 | 0.74 | 0.88 |
| Global hardness (η) | 2.85 | 2.83 | 2.84 |
| Ionization potential (I) | 6.57 | 6.42 | 6.56 |
| Electronegativity (χ) | 3.72 | 3.58 | 3.72 |
| Electrophilicity (ω) | 2.42 | 2.26 | 2.43 |
